# Can Even a Small Amount of Greenery Be Helpful in Reducing Stress? A Systematic Review

**DOI:** 10.3390/ijerph19169778

**Published:** 2022-08-09

**Authors:** Jiaqi Gu, Haixiao Liu, Hong Lu

**Affiliations:** 1 School of Urban Design, Wuhan University, Wuhan 430072, China; 2 School of Carey Business, Johns Hopkins University, Washington, DC 20036, USA

**Keywords:** green space, green scale, plants, stress relief, health

## Abstract

A positive experience of nature triggers beneficial mental and physical responses. Today, we live in a rapidly urbanizing world where access to nature is often limited. Against this backdrop, this systematic review investigated studies on the effectiveness of small-scale greenery for stress reduction. We searched EMBASE, Cochrane, Web of Science, Scopus, PubMed, and Science Direct, searching databases from inception to April 2022. Studies were screened against predetermined criteria, and the risk of bias was assessed using the Cochrane Handbook for Systematic Reviews of Interventions for RCTs and The Risk of Bias in Non-Randomized Studies of Interventions (ROBINS-I) tool. Of the 2500 records identified, we screened 1817 citations for eligibility, which included 13 RCT studies and 6 non-RCT studies. The studies were conducted in eight different countries. The study populations included office workers, students, senior citizens, and patients with specific diseases. Research has mainly focused on indoor greening, with relatively little research on small-scale outdoor greening. All included studies assessed the impact of the intervention on various stress reduction-related outcomes, with the most common stress measures being blood pressure and the State Trait Anxiety Inventory (STAI). Various beneficial effects of the interventions on human health were reported in all 19 studies, 15 of which reported positive effects on stress reduction. All included studies were at high risk of bias. It is recommended that future studies in this area take appropriate measures to reduce bias and improve quality in order to build a strong evidence-based medical foundation. According to our findings, even very small-scale greening, including indoor green walls and potted plants, may provide effective help for stress relief. Understanding the physiological and psychological benefits of small-scale greenery can help better provide more opportunities for urban residents to engage with nature in the context of dense urban trends, as well as provide some reference for urban design planning.

## 1. Introduction

Human health and well-being have always been affected by the quality of the environment in which people live [1]. With urbanization, a large percentage of the population lives in stress-filled environments [2]. Previous research has found that people who grow up in cities have higher rates of psychiatric disorders, anxiety disorders, and depression than those who grow up in rural areas [3]. These disorders are often associated with or triggered by high levels of stress [4]. Symptoms of stress may include decreased memory and concentration, insomnia, increased heart rate, headaches, and muscle aches and pains [5]. The consequences of these symptoms may include decreased functioning socially and at work. Chronic stress can suppress the immune system and trigger cardiovascular disease, stroke, depression, asthma, and other serious health problems [6].

Numerous studies have shown that positive experiences in nature trigger beneficial psychological and physiological responses, such as lower blood pressure and heart rate, reduced muscle tone, better concentration, lower stress hormone levels, and improved creative problem-solving skills [7,8,9,10,11,12]. The effects of exposure to nature on restorative benefits have been explored differently, with two major theories coming from an environmental psychology perspective: attention recovery theory (ART) and stress reduction theory (SRT). The attention recovery theory proposes that the natural environment provides a “soft charm” that enables people to concentrate effortlessly [8,13]. Stress reduction theory suggests that the presence of nature brings about evolutionary psychological responses related to safety and survival. Hence, exposure to nature activates our parasympathetic nervous system and promotes recovery from psychophysiological stress [14].

However, as cities continue to grow and densify, it becomes increasingly difficult to engage with nature in urban environments [15,16,17,18]. Public green space in compact cities is very limited due to geography and population [12,19,20], and cities once characterized by extensive greenery are transforming into more compact and dense. Large urban green spaces are often encroached upon or fragmented, or accessible nature is pushed outside the city limits [21,22]. People living and working in urban high-rise buildings receive limited opportunities to view urban nature and parks [16]. According to the National Human Activity Pattern Survey (NHAPS), people spend almost 90% of their time indoors (Klepeis et al., 2001), which indicates a further disconnection from nature [23]. At the same time, unfortunately, worldwide COVID-19 control efforts have limited outdoor activities for the general public, as well as various recreational activities [15]. For example, the Chinese government has issued travel bans, lockdowns, and home orders, group gatherings have been canceled, and non-essential employees have been required to work from home. Various traffic control measures have been taken throughout China to contain the spread of the epidemic, and people’s travel patterns and range of travel have been affected [16,17]. People in cities have difficulty leaving the city to visit large areas of nature. Social pressure becomes very difficult to relieve [15].

It has been found that more frequent contact with green space brings benefits and that natural distance affects the frequency of use, with greater distance meaning less frequent use [12,19]. As a result, cities have had to resort to methods of expanding urban greenery through small-scale green features [18]. The interaction between people and nature is increasingly dependent on the quality of the landscape outside the formal green space network [24], such as pocket parks, green roofs, etc. In built environments where people lack contact with nature, indoor plants have also been shown to reduce stress and improve people’s subjective well-being [25]. Small-scale green has many advantages, such as high accessibility, convenience, attractiveness, and low cost, both indoors and outdoors, providing convenient and attractive green exposure to urban residents and meeting the demand for people to benefit from “green” [26,27,28,29].

Although there have been previous meta-analyses and systematic evaluations of green environments for stress reduction, these studies have focused on large green spaces, such as forests and large parks, and lack systematic evaluations of small-scale green features, such as pocket parks, community green spaces, private gardens, and even single trees and decorative bonsai. The primary objective of this study was to synthesize evidence on the effectiveness of small-scale greening as an intervention in reducing physical and psychological stress levels and promoting people’s physical and mental health.

## 2. Materials and Methods

We follow the Preferred Reporting Items for Systematic Reviews and Meta-Analyses (PRISMA) guidelines for reporting this systematic review.

### 2.1. Search Strategy

We conducted electronic searches of EMBASE, Cochrane, WOS, Scopus, PubMed, and Science Direct. Databases were searched from inception to April 2022. Search terms are related to small green (such as “small-scale green”, “pocket park”, “green roof”, or “home garden”) and stress reduction (such as “stress reduction”, “stress-related”, “blood pressure”, or “mental health”). There were no restrictions on the year of publication. All geographical regions were eligible, but only references written in English were included. Detailed search strategies are shown in the Supplementary Materials.

### 2.2. Inclusion and Exclusion Criteria

Inclusion criteria were defined according to PICOS (Population, Intervention, Comparison, Outcome, and Study Type) policies. For the population (P), namely the participants under consideration, any adult was eligible regardless of their physical or mental health status. For intervention (I), the experimental group was a small-scale greening. Small-scale greening was defined as green facilities less than 0.4 ha in size, including outdoor greening, such as green roofs, pocket parks, private gardens, and street trees, and also small-scale green features, such as indoor green walls, decorative planters, and greenery (virtual environments were also considered) [18,30,31,32]. For (C), no greening or any other comparative intervention within the environment. The primary outcome (O) was stress reduction. This should be a quantitative measure. The measure must refer to current physiological stress responses, as well as self-reported psychological indicators (e.g., mood, anxiety, distress, perceived stress, recovery, attention, or cognitive functioning). (S) Inclusion criteria were limited to experimental clinical studies with interventional factors. Subjects were categorized into randomized controlled and non-randomized controlled studies based on whether or not they were randomized, and observational studies without intervention factors were not included. As shown in Table 1.

### 2.3. Study Selection

Records from each database were downloaded and merged into Endnote X9. Duplicates were removed, and titles, abstracts, and full text were filtered. Study selection was conducted in three stages: the first stage involved screening the titles of the articles against the inclusion criteria; the second stage involved screening the abstracts; the third stage involved a full screening of the articles. In the first and second phases, screening was deferred to the next phase if there was doubt about the inclusion of any article. At this point, all team members were involved in discussions to resolve the issue. If there were duplicate studies, the authors randomly selected one from three databases.

### 2.4. Data Extraction

The first author (Jiaqi Gu) used a standardized form to extract the following information from each of the included studies:

Article identifier (author name, year of publication, country); study identifier (setting, sample size, design); purpose of the study; and results. Results were extracted into a coding frame using Microsoft Excel, synthesized, and tabulated. A second researcher (Haixiao Liu) checked all data extraction tables, and disagreements were resolved through discussion until consensus was reached.

### 2.5. Quality Assessment

Each author independently assessed the risk of bias for each of the included studies. For the RCT, the risk of bias was assessed using the criteria outlined in the Cochrane Handbook for Systematic Reviews of Interventions [33]. The following areas were assessed against the Cochrane Risk of Bias Checklist: random sequence generation (selection bias), allocation concealment (selection bias), blinding of participants and personnel (performance bias), blinding of outcome assessment (detection bias), incomplete outcome data (attrition bias), selective reporting (reporting bias), and other bias performance. The risk of bias was assessed for each study-related entry. The risk of bias in each domain was divided into three categories: low risk, high risk, or unclear risk of bias.

The Risk of Bias in Non-Randomized Studies of Interventions (ROBINS-I) tool was used for non-randomized studies [34]: confounding, participant selection, classification of interventions, deviation from intended intervention, missing data, measurement of outcomes, reporting bias, and overall bias. The risk of bias in each area was divided into five categories: low risk of bias, moderate risk of bias, serious risk of bias, critical risk of bias, and no information.

Any differences are resolved through discussion.

## 3. Results

### 3.1. Search Results

**Figure 1:**
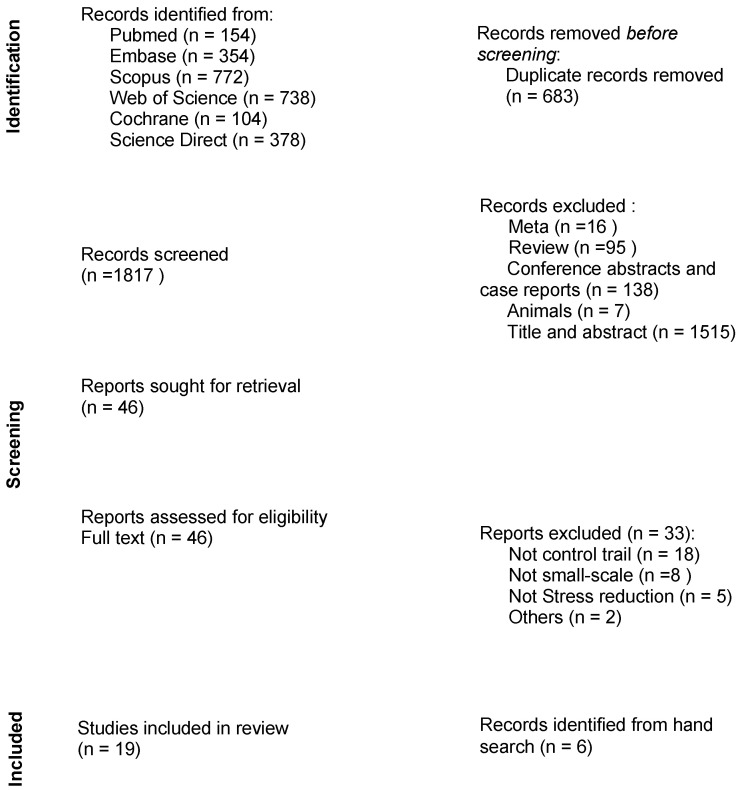
Flow diagram of the study selection process.

The key characteristics of each included study are detailed in Table 2. The initial search of the six databases produced a total of 2500 results. After removing 939 duplicate and substandard studies, a total of 1561 titles and abstracts were screened. A full-text search was also conducted for papers considered potentially relevant. The review flow chart is detailed in Figure 1.

The range of publication years for the included studies was from 1995 to 2021, with the majority of studies after 2010 (*n* = 13). These included studies were conducted in eight different countries. Ten of the nineteen studies were conducted in Asia, three in Europe, five in North America, and one in Australia. The countries with the highest frequency of inclusion were China and the USA, with ten studies (five studies each). Most studies were randomized controlled studies (*n* = 13) and the remainder were non-randomized controlled studies (*n* = 6) [5,12,16,35,36,37,38,39,40,41,42,43,44,45,46,47,48,49,50].

The size of the sample surveyed varied considerably, with sample sizes ranging from 15 [38] to 240 [12]. Fifteen of these studies had a sample size of no less than 50. Participants were generally young, with 84% of the studies (*n* = 16) recruiting students (mean age < 30); two studies were working people and patients with a wide age range [37,48]; one study recruited senior citizens (range 61–97 years) [38]; and one study had identified a specific population: patients recovering from appendectomy [48]. Each of the nineteen studies had either male or female participants [45,46]. Fourteen articles had both male and female participants. Three studies did not describe the gender of the participants [39,44,47].

### 3.2. Risk of Bias

**Figure A1:**
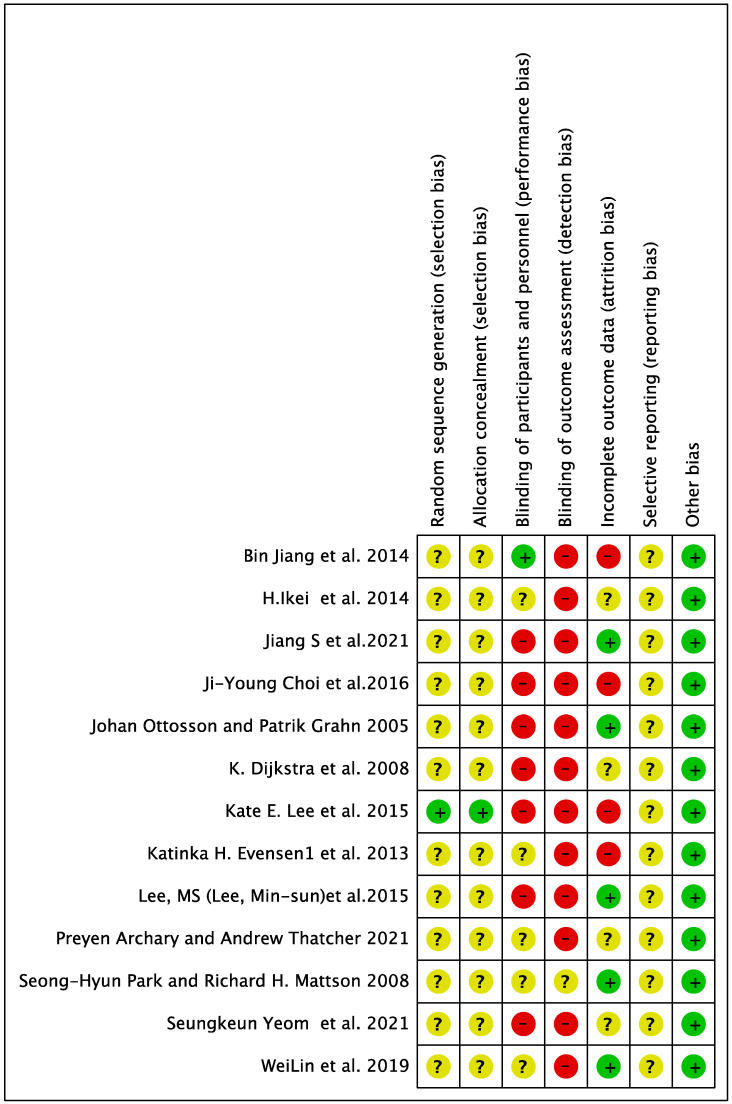
Risk of Bias table for included RCT studies. Bin Jiang et al., (2014) [5], H. Ikei et al., (2014) [40], Jiang S. et al., (2021) [45], Ji-Young Choi et al., (2016) [50], Johan Ottosson and Patrik Grahn (2005) [38], K. Dijkstra et al., (2008) [36], Kate E. Lee et al., (2015) [35], Katinka H. Evensen1 et al., (2013) [42], Lee, MS (Lee, Min-sun) et al., (2015) [46], Preyen Archary and Andrew Thatcher (2021) [41], Seong-Hyun Park and Richard H. Mattson (2008) [48], Seungkeun Yeom et al., (2021) [16], WeiLin et al., (2019) [12].

**Figure A2:**
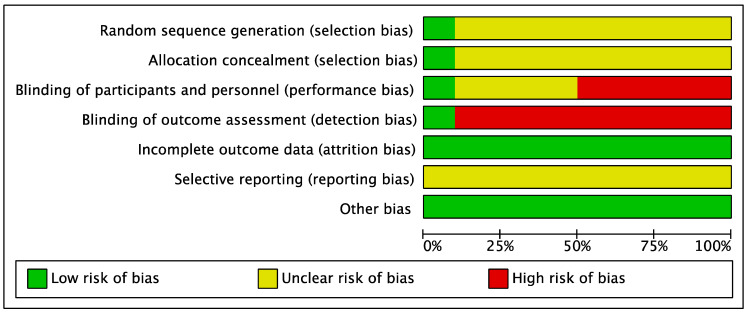
Risk of Bias graph for included RCT studies.

Thirteen randomized studies were evaluated using the standard [33] tools outlined in the Cochrane Handbook for the Systematic Evaluation of Intervention Systems, and six non-randomized studies were evaluated using ROBINS-I. (The overview of the risk of bias in the included studies is presented in Appendix A, Figure A1 and Figure A2, and Table A1).

Of the thirteen randomized studies included, only one described their method of random assignment and was therefore designated as low risk of bias in this domain. The remaining studies did not describe their method of random sequence generation and were rated as unclear [35]. In terms of blinding, one study noted a double-blind trial [5]. Other RCTs in this review either did not provide information on participants and personnel or lacked blinding of participants and personnel and were assessed as unclear or at high risk of bias in this area. There were no participant dropouts in the five randomized studies, complete data were reported [12,38,45,46,48], and therefore the risk of bias was low. Four randomized studies with missing data were judged to be at high risk of bias [5,35,42,50]. The remaining randomized studies had incomplete information, so it was difficult to judge the completeness of the data, or the studies did not mention completeness issues and were therefore designated as unclear risk.

Of all included non-randomized studies, five were flagged as having an overall significant risk of bias. In terms of confounding factors, the four included in non-randomized studies were flagged as having a serious risk of bias, and many important confounding factors were identified (weather, food, physical activity, whether smoking and drinking alcoholic or caffeinated beverages before the experiment; study and homework stress; and plant wilt, etc.). For example, in the study by Ke-Tsung Han (2008) et al., participants may have been influenced by confounding factors such as exam stress and homework [33]; in the study by Virginia I. Lohr et al., (1996), participants’ level of human–computer use and keyboarding skills were unclear, and thus both were noted as having a serious risk of bias [49]. One study was marked as moderately biased because participants were controlled for physical status as well as dietary intake [39].

Another study provided too little information on confounding factors and was marked as “no information” [47]. In terms of participant selection, all non-randomized studies were judged to have a low risk of selection bias due to the selection of participants in the included studies independent of the intervention or outcome. Judging by the character of the intervention, the risk of misclassification of intervention and control sites is low. Only one study was considered as a moderate “deviation from the intended intervention” because participants experienced unexpected plant death and the need to replace plants during plant care [37]. For the missing data domain aspect, four participants in one study were rated as having a serious risk of bias because they did not complete their study tasks resulting in missing data and were not addressed by appropriate analysis [44]. All other non-randomized studies had complete data or dealt with missing data and were judged to have low-risk bias. In terms of bias in the measurement of outcomes, all studies had data obtained through self-reporting, four studies were identified as having a severe risk of bias, and two studies were identified as having a moderate risk of bias [37,39]. All other non-randomized studies were at moderate risk of reporting bias due to reporting of complete data but no associated study protocol.

### 3.3. Characteristics of the Interventions

The duration of plant exposure ranged from 40 s [35] to 2.5 months [43], most commonly less than 15 min (*n* = 13). Four tasks were multi-day exposures [28,43,44,48], and two studies did not specify the duration of exposure [36,49]. There were nine studies with interventions in which participants simply had to view greenery or photographs [5,16,35,36,39,40,41,42,50]. Seven study participants performed personal activities in green spaces or spaces containing green [12,38,43,44,45,48,49]. Participants in three studies performed plant care or transplantation-type interactions [37,46,47].

The most frequently used measure was blood pressure (*n* = 8). It was followed by the State Trait Anxiety Inventory (STAI, *n* = 6). Pulse rate (*n* = 5), electroencephalogram (EEG, *n* = 5), semantic differential (SD, *n* = 5), and heart rate variability (HRV, *n* = 4) were also used. Dundee Stress State Questionnaire (DSSQ-S), Finger SpO2 (fingertip oxygen saturation), Zuckerman Inventory of Personal Reactions (ZIPER), the Stress Arousal Checklist, Electrodermal activity (EDA), Trier Social Stress Test (TSST), Salivary Cortisol, Skin Conductance, Skin Temperature, 101-point numerical rating scale (NRS-101), Likert scale, Restorative Component Scale (RCS), and Restorative Scale (RS) were used once.

Most studies were indoor greenery (*n* = 15), and others were small outdoor green spaces (*n* = 4). A small number of studies were greened for virtual scenes (*n* = 3) and scene photos (*n* = 2). In terms of environmental exposure, most studies (*n* = 15) did not have any green within the control environment. Two studies were controlled for environmental exposure to different areas of greenery [16,50], one study was controlled for different greenery behaviors and per capita area within a small green space [12], and one study was controlled for the presence or absence of odor from greenery [45].

**Figure 2:**
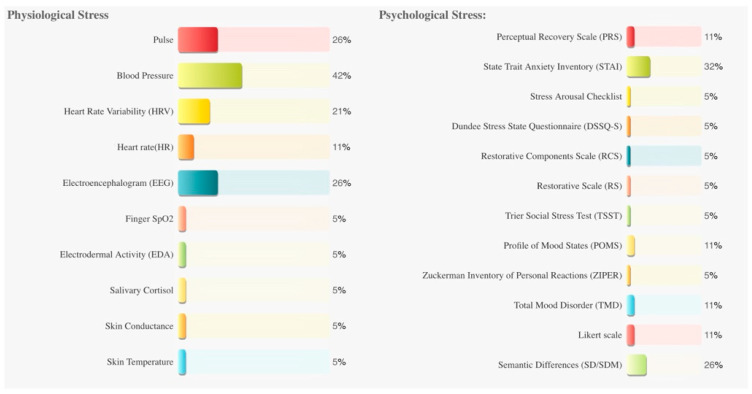
The measurement methods and frequencies of the included studies.

The measurement methods and frequencies of the included studies are shown in Figure 2.

### 3.4. Intervention Results

#### 3.4.1. Effect on Blood Pressure

Five RCT studies [12,38,45,46,48] and three non-RCT studies [39,47,49] reported on the effects of small-scale green on blood pressure. An increase in blood pressure indicates an increase in stress [38]. Six studies showed a decrease in blood pressure and a decrease in stress in participants through interventions [12,45,46,47,48,49]. One study showed no significant change in blood pressure for 5 min of ornamental plants in an indoor work environment [39]; another study on senior citizens in a small garden showed that one hour of outdoor recreation did not affect participants’ blood pressure [38]. WeiLin et al., found no significant differences in blood pressure between the experimental groups after a green space behavior and per capita area intervention in six small green spaces and that the positive effect of the green environment on blood pressure was dynamic, with positive effects appearing in the first few minutes, then decreasing and disappearing in the long term [12].

#### 3.4.2. Effect on Pulse Rate

The lower the pulse rate in the normal range, the less physiological tension and stress there will be [12]. Five studies included pulse as an assessment metric in intervention trials [12,37,40,45,49]. Studies have reported that both small outdoor green spaces and small indoor plants are conducive to lower pulse rates. WeiLin et al., claimed that walking in high PCA (per capita area) and sitting in low PCA had the most powerful effect on reducing stress.

#### 3.4.3. Effect on HRV

Heart rate variability quantifies changes in heart rate associated with internal versus external environmental changes, particularly those due to autonomic nervous system activity. It is an indirect measure of mental stress and a key indicator of physiological stability [50]. Four RCT studies [16,40,46,50] reported the results of small-scale green for participant heart rate variability. In the study of the physiological and psychological effects of green walls on occupants, Seungkeun Yeom et al., concluded that heart rate variability is more sensitive to noise waves, and the measurement time is shorter and less reliable, thus discharging this indicator from the analysis. A report from 85 secondary school students showed that foliage plants resulted in a significant increase in parasympathetic activity (high-frequency component), suppression of sympathetic activity (low-frequency component), a significant decrease in pulse rate, and greater comfort and relaxation for the students upon seeing the plants [40]. Lee, MS (Lee, Min-sun) et al., claimed that the average activity in HRV (equivalent to sympathetic activity) decreased at the end of the vegetative task. Ji-Young Choi et al., reported that there was no significant difference in heart rate variability parameters among participants under conditions of 5%, 20%, 50%, and 80% green indices in indoor spaces; and that even very low green color provided physiological and psychological benefits [50].

#### 3.4.4. Effect on Heart Rate

An RCT study reported a heart rate lowering effect of ward flowering foliage plants on patients undergoing abdominal surgery, with a lower heart rate implying a more positive physiological response [48]. A non-RCT study on senior citizens reported no effect of one hour of outdoor recreation on heart rate [38].

#### 3.4.5. Effects on EEG

In this review, a total of five studies [16,39,45,47,50] included EEG as a basis for stress reduction assessment in their experiments. Generally, it is believed that alpha and beta waves show the closest relationship out of human emotions, with high alpha waves strongly associated with relaxation and high beta waves associated with attention and alertness [45]. Among the included studies, four of the RCT studies reported that exposure to small-scale green showed an increasing phenomenon of alpha and beta wave mean values on participants’ EEG, but Seungkeun Yeom et al., showed a decrease in alpha and beta wave mean values under large green wall conditions, adding mental stress and fatigue as well as anxiety levels. A non-RCT study showed positive changes in EEG measurements after a small-scale greening intervention in indoor spaces, with no significant differences in EEG data between the different green indices, but there were gender differences, with men having significantly higher EEG power than women [50].

#### 3.4.6. Effects on Anxiety

Two RCT studies [16,48] and four non-RCT studies [37,39,43,47] assessed the effects of small-scale greening on participants’ status and anxiety levels. Five of the studies had significant reductions in STAI scores after the intervention [16,37,39,47,48], indicating that exposure to small-scale green was associated with significant improvements in anxiety levels and relief of psychological stress. A study was conducted on a specific population: post-appendectomy patients, and it showed that in-room plants were beneficial for patients’ post-operative recovery. Seungkeun Yeom et al. found that the improvement effect of a large indoor green wall was much lower than that of a small green wall, leading to only a small decrease in state anxiety. In Ke-Tsung Han’s study, the improvement in the anxiety state by six pots of leafy plants in the classroom was immediate, diminished with time, and statistically insignificant over a long period.

#### 3.4.7. Effect on Emotion

Small amounts of greenery have a significant restorative effect on mood. Contact with greenery can be comforting [40,45,46,47,50], natural and relaxing, and even small amounts of greenery can have a relaxing effect on people [50]. The Total Mood Disorder (TMD) and Profile of Mood States (POMS) measures found that participants gained more positive emotions and fewer negative emotions, with overall mood showing a more beneficial state [12,45]. One study showed that participants’ Well-Being Measure (WBM) decreased over time [43].

#### 3.4.8. Impact on Restorative Outcomes

Three studies reported a restorative effect of small-scale green interventions on participants [35,41,43], with Preyen Archery and Andrew Thatcher claiming a positive effect of the presence of houseplants on emotional recovery and no effect on cognitive recovery [41]. While another study concluded that there was no significant restorative effect of living plants and that visual richness in the environment may be a restorative factor [42].

#### 3.4.9. Effects on Attention

Studies have reported that green roofs (virtual), outdoor gardens, and small plants in computer labs have an attention-improving effect [35,38,49]. However, in the Virginia I. Lohr et al. study, the specific exposure time was not stated, depending on the computer task completion time. WeiLin et al., claimed that six small green spaces had no significant improvement effect on college students’ attention [12].

#### 3.4.10. Impact on Self-Reporting

In one study, by having participants view photographs of a ward with or without plants, it was shown that indoor plants reduced participants’ self-reported stress by increasing the attractiveness of the room [36]. In another study, it was shown that a 6-min plant viewing intervention increased participants’ task engagement and reduced distress and worry [41].

#### 3.4.11. Impact on Others

Thermal biofeedback is a method used to assess pressure by measuring the skin temperature of the fingers or toes. Cammie K. Coleman and Richard H. Mattson showed an increase in skin temperature during the first 5 to 8 min of a 20-min thermal biofeedback session, indicating stress reduction [44]. Another study aimed at patients recovering from surgery found that flowering plants in the ward had a relieving effect on patients’ postoperative pain through a 101-point numerical rating scale (NRS-101) that assesses anxiety and fatigue [48].

### 3.5. Intervention Effectiveness

Of the 19 included studies, 84% (*n* = 16) reported varying degrees of reduction in participants’ stress in the intervention condition. Ninety-five percent (*n* = 18) of the studies with small-scale greening did not report negative effects. Only one study reported a negative effect of small-scale greening: Seungkeun Yeom et al. 2021 reported that a large green wall increased participants’ stress, fatigue, and anxiety levels [16]. Interventions and effectiveness results are shown in Table 3.

#### 3.5.1. Effectiveness of Indoor Small-Scale Greening for Stress Reduction

Seven studies [39,40,41,42,45,49,50] evaluated the stress-reducing effects of short exposure (no more than 10 min) to small-scale indoor greenery. The presence of plants elicited significant changes in brain activity [39]. By measuring physiological stress such as blood pressure, pulse, and EEG, as well as psychological stress such as STAI and SD, the results of five of these studies showed a significant stress-reducing effect of small-scale greenery. Both indoor viewing greenery [39,40,45,50] and performing computer tasks in a small-scale greening environment [49] were beneficial for stress relief. Not only that, but participants also feel more relaxed and comfortable in a green environment [40] and even have increased productivity [49]. Two studies [41,42] did not report a stress-relieving effect of indoor greenery on participants’ psychological stress. In addition, due to the lack of measurement of physiological stress, it was not possible to determine whether the interventions affected physiological stress.

The results of the three included studies [37,43,48] showed that prolonged (more than 1 day) exposure to small-scale indoor greenery had a mitigating effect on participants’ psychological stress. Except for the study by Ke-Tsung Han (2008), which lacked a measure of physiological stress [43], the remaining two studies showed a significant reduction in physiological stress under small-scale greenery conditions [37,48]. Masahiro Toyoda et al. (2019) claimed that conscious gazing at nearby plants can reduce psychological and physiological stress in office workers [37]. Seong-Hyun Park and Richard H. Mattson (2008) showed a significant reduction in postoperative analgesic intake through an intervention in which patients exhibited more positive physiological responses (lower systolic blood pressure and heart rate) and more positive feelings [48].

A total of two studies [46,47] assessed the effects of plant activities on stress in subjects. Intervention results from both studies showed that a 15-min long plant transplant activity had a significant ameliorating effect on participants’ psychological stress. Lee, MS (Lee, Min-sun) et al. (2015) concluded that positive interactions with indoor plants can reduce both physiological stress and psychological stress [46]. This is achieved by suppressing sympathetic nervous system activity and diastolic blood pressure as well as promoting a sense of comfort, soothing, and naturalness. The results of another study suggest that contact with plants can minimize mental stress [47].

#### 3.5.2. Effectiveness of Small-Scale Outdoor Greenery on Stress Reduction

Of the 19 included studies, a total of two studies [12,38] assessed the effects of small-scale outdoor greenery on stress. With six outdoor small-scale simulated green spaces, WeiLin et al., (2019) reported that walking in high PCA (per capita area) and sitting in low PCA had the greatest reduction in stress [12]. Another study showed that senior citizens in a home for the elderly relaxing in an outdoor garden for one hour had only a restorative effect on attention. There was no effect on physical stress, while data on the measurement of psychological stress were lacking. Thus the small-scale greening in this study had no stress-reducing effect [38].

#### 3.5.3. Effectiveness of Non-Living Plant Environments on Stress Reduction

A total of two studies [16,35] had small-scale greening as a virtual scenario. Both studies reported that small-scale greenery had a reducing effect on participants’ stress to varying degrees. One of the studies, Seungkeun Yeom et al., (2021), showed a substantial reduction in stress levels in subjects with small indoor green wall conditions. However, when the indoor green wall was larger, it increased the mental stress, fatigue, and anxiety levels of the subjects [16]. Subjects in another study had elevated subcortical arousal and cortical attentional control after micro-breaks from viewing a simulated view of a green roof. The restoration of attention brought benefits, including improved performance and mood, as well as reduced stress [35].

A study by Bin Jiang et al. found that viewing small-scale greening videos reduced stress (2014). The intervention condition’s measures of physical and psychological stress revealed a significant effect of community street tree cover density on stress reduction. However, the effects of different tree cover densities on stress reduction differed significantly by gender. Males’ stress recovery dose curves showed an inverted U-shape, with faster stress recovery at 1.7–24% tree cover density, no significant change at 24–34%, and slower recovery above 34%, with moderate tree density causing greater stress reduction. Furthermore, there was no significant difference in pressure recovery for different tree densities among females [5].

Out of the 19 included studies, two showed an attenuating effect of plant pictures on stress [36,44]. According to one study, when people saw pictures of hospitals with plants, their levels of stress were lower than when they saw pictures of hospitals without plants. Indoor plants eased the tension by making the space more appealing [36]. Another study used plant color photography or live plants for the intervention. According to the study’s findings, 38% of participants responded favorably to both actual plants and photos.

## 4. Discussion

In this systematic review, 19 studies from eight countries were synthesized. These studies investigated the impact of small-scale greening (photos, videos, or virtual environments) on stress relief.

Most studies use a holistic approach, collecting subjective psychological and objective physiological data to assess stress status. Pulse, blood pressure, HRV, EEG, STAI, and SD/SDM were frequently used to assess stress in 19 studies. Psychological stress is measured by subjective rating scales such as the STAI; physiological stress is usually measured by this analysis due to the validity, reliability, and ease of collection of pulse, blood pressure, HRV, and EEG data.

Our findings suggest that all of the included studies reported that varying degrees of small-scale greening are beneficial for health. Most of the studies (*n* = 15) demonstrated the mitigating effect of small-scale greening on physical or psychological stress as measured by various indicators. At the same time, we found that the intervention produced beneficial results such as restored concentration, pain relief, and feeling more comfortable and natural. Of the literature included in this review, a small number of studies reported interactions with plants, such as plant transplantation [46,47] and care [37], nine studies for directed attention to greenery, and seven studies for direct exposure to environments with greenery [12,38,43,44,45,48,49]. The results of the study found that all three intervention types were helpful for stress relief, and due to the different number of included literature and insufficient data, it was not possible to determine which type was most helpful for stress reduction. However, it is unclear whether uncontrollable factors such as plant wilting during plant care can adversely affect stress reduction and needs to be studied in depth through other literature. An included study on primroses found that scented primroses were more effective in reducing stress than unscented ones. The authors concluded that the effect of plants on people is not only in the visual perception but that plants with aromatic properties can induce physiological and psychological relaxation through olfactory channels [45].

The immediate effect of plant stress reduction was significant. Studies have found significant stress reduction effects for short periods of exposure to small-scale green environments, consistent with the positive physiological responses to plants described in previous studies over 3 to 6 min [37,44]. Some studies concluded that prolonged exposure to stress, although improved, has a statistically insignificant effect [43].

The stress-reducing effect of greening facilities is not proportional to their size. When the indoor green wall is too large, it not only loses its effectiveness in reducing stress but also has a negative psychological impact. A small outdoor green space study included in this systematic evaluation showed the strongest effect in reducing stress when walking in high PCA (per capita area) and sitting in low PCA, but there is a lack of relevant data on whether other experimental settings are effective in stress reduction. Stress reduction theory suggests that the presence of a natural environment (restorative environment) brings about a psychological, evolutionary response related to safety and survival and therefore generates positive emotions. However, larger areas of green space are not our only goal, and health benefits can only be maximized when the area of green space per capita is matched with appropriate green space behavior. In the planning and design of urban green spaces, designers should provide more appropriate green space structures and layouts and advocate addressing the uneven distribution of urban green spaces rather than blindly pursuing larger green spaces [12,16,51].

A lower greening index and very small-scale greening may also have a positive impact on stress reduction. Ji-Young Choi et al. found no significant differences in physiological parameters among the four (5%, 20%, 50%, and 80%) green index levels studied, although participants subjectively preferred a 50% indoor green index. Another study found that stress recovery was faster when tree cover density was between 1.7% and 24% and that tree density above 24% was associated with slower recovery times, but the results were limited to males. For women, there was no relationship between different densities of tree cover and stress recovery. Studies have reported finding that even small, single indoor plants can people exert stress-reducing effects during conscious or unconscious gaze [37,39,50].

Outdoor green space is not the only thing that works for stress relief. Indoor greenery is another important way for people to get in touch with nature. For people who have less time to get in touch with outdoor green spaces and parks, contact with indoor plants can effectively reduce stress and improve work efficiency. Green facilities in hospital wards can bring more positive physical and psychological feelings, reduce pain, and relieve psychological stress for patients recovering from some diseases after surgery [36,37,39,42,43,46,47,48,49].

The heterogeneity of the studies included in this review is high. Due to the nature of the studies, participants were likely aware of the hypotheses being tested. Most studies, therefore, showed a high risk of bias in terms of blinding, and there is no way to avoid some subjectivity in the results. In addition, some studies [32,38,43] were of long duration, and many uncontrollable factors may have influenced the experimental results. In order to strengthen the clinical evidence base, it is advised that future studies in this field use more stringent and standardized procedures to reduce bias and increase quality. This will help convince policymakers and health professionals that even small-scale greening can have a beneficial impact on stress mitigation.

## 5. Limitations

Our review has several limitations. The keywords used in our search strategy for identifying eligible studies may have been narrow due to the lack of a standard definition of small-scale greening and inadvertently excluded greening that may have met the inclusion criteria size. Most of the exposure environments in the included literature were indoor environments, and data from studies of small-scale outdoor greening were lacking, so the findings lacked greater rigor and accuracy. There is a wide variety and poor uniformity of methods regarding pressure testing in the included literature. Therefore, a relevant meta-analysis could not be performed. Many studies suffer from small sample sizes and single study populations, and the results are not generalizable. Most of the included studies were single experiments with an intervention length of 15 min or less, and the findings could only demonstrate the effect of greening in a limited time task, and it was not possible to determine whether the same effect on stress existed with long-term exposure. A small number of exposures to non-living plant environments (photographs and virtual scenes) are also included in this review, and although supported by relevant research theories, their ability to replace real environments remains controversial. Soil and water from indoor plants are potential sources of bacteria, and we cannot rule out the risk of harmful health effects from indoor landscaping and the impact of airborne particles produced. There are many uncontrollable factors during long experiments, such as academic stress, diet, sleep, etc., resulting in a lack of rigor in the results. The included study populations were mostly young students, which limits the generalizability of our findings. In addition, the research literature in this review is all English-language studies, which should be screened more comprehensively.

## 6. Conclusions

In this systematic review, 19 studies from 8 countries were synthesized. These studies investigated the impact of small-scale greening (photos, videos, or virtual environments) on stress relief.

Although more research exists on the benefits of natural environments for stress reduction, there is a lack of systematic research on the role of small-scale greenery in reducing stress. This systematic evaluation helps to fill this knowledge gap and suggests that small-scale greenery, including green walls and potted plants, may have beneficial effects on stress reduction. Understanding the physiological and psychological benefits of small-scale greening could help better inform urban design planning in the context of dense urban trends that provide more opportunities for urban residents to engage with nature. However, the research is highly biased and of low quality, and more rigorous studies are needed to improve our understanding of the relationship between greening and stress reduction.

## Figures and Tables

**Table 1:** Inclusion and exclusion criteria.

Inclusion Criteria:	Exclusion Criteria:
Empirical studies.	Studies that do not look at empirical evidence.
Studies examined the association between small-scale greening and physiological stress responses, as well as self-reported psychological indicators (e.g., mood, anxiety, distress, perceived stress, recovery, attention, or cognitive function).	Studies did not examine the association between exposure to small-scale greening and physiological stress responses, as well as self-reported psychological indicators (e.g., mood, anxiety, distress, perceived stress, recovery, attention, or cognitive function).
Studies that use human participants	Studies that do not use human participants
Records were not written in English.	Records were not written in English.

Reprinted with permission from Ref. [6]. 2022, Yao W. et al.

**Table 2:** Basic characteristics of the studies included in the review.

Authors and Year of Publication	Country	Design	Sample	Setting(s)	Intervention	Results
A. Hassan et al., (2020) [39]	China	Non-randomized control trials	N = 50 (No gender information) Mean age: 20.3, SD = 16.3 Chinese students from the campus of Sichuan Agricultural University	Greenery included: Indoor with ornamental plants (experimental) No greenery: Indoor without ornamental plants (control)	5 min of viewing	The STAI scores were lower in the presence of plants, and there was no significant difference between blood pressure and pulse rate. Observation of plants in the work environment enhanced brain wave activity and decreased anxiety to reduce mental stress.
Bin Jiang et al., (2014) [5]	USA	Randomized controlled trials	N = 158 (80 males and 78 females) Mean age: 21.2, SD = 2.7 Healthy Adults	Video of 10 neighborhood street scenes with different vegetation densities	Watch the 6-min video	There were significant differences between males and females: tree cover at different densities was not associated with pressure recovery for females. For males, pressure recovery increased at 1.7–24%, no change at 24–34%, and slower recovery above 34% (inverted U-shape). Moderate tree density caused greater pressure reduction.
Cammie K. Coleman and Richard H. Mattson (1995) [43]	USA	Non-randomized control trials	N = 50 (No gender information) Age: 18–34 (No average age information) College Students	Greenery included: A green plant in the room or a life-size color photo of that plant (experimental) No greenery: A metal stool in the room (control)	Participated in 20-min sessions twice a week for 6 weeks.	Live plants and photographs had a positive response for 38% of participants. 23% had reduced stress in the control group. No significant differences were found for the rest.
H. Ikei et al., (2014) [40]	Japan	Randomized controlled trials	N = 85 (41 males and 44 females) Mean age: 16, SD = 0.9 High school students	Greenery included: Exposed to foliage plants (experimental) No greenery: No foliage plants (control)	Ornamental plants for 3 min	Foliage plants resulted in a significant increase in parasympathetic (high-frequency component) activity, suppression of sympathetic (low-frequency component) activity, and a significant decrease in pulse rate. More comfort and relaxation after seeing the plants.
Hassan, Ahmad, et al., (2018) [47]	China	Non-randomized control trials	N = 50 (No gender information) Mean age: 19.6, SD = 1.42 College students experiencing high academic stress	Greenery included: Indoor plant transplantation (experimental) No greenery: Playing mobile app games (control)	15 min plant transplanting work	Blood pressure decreased significantly. No change in pulse rate. STAI decreased significantly. SDM felt more relaxed. Alpha and beta wave averages increased over time during the transplantation task. The results of the study suggest that contact with plants minimizes mental stress.
Jiang S. et al., (2021) [45]	China	Randomized controlled trials	N = 50 (all females) Mean age: 22.32, SD = 2.56 Female college students	Scented primroses (experimental) Unscented primroses (control)	Exposure to plant environment for 10 min	Mean blood pressure and pulse rate in both conditions decreased significantly after the experiment, and the mean EEG was higher. Both groups of primroses were psychologically and physiologically beneficial. Fragrant primroses caused better effects.
Ji-Young Choi et al., (2016) [50]	Korea	Randomized controlled trials	N = 103 (51 males and 52 females) Mean age: 21, SD = 2.3 College students	Green indices for indoor spaces were 5%, 20%, 50%, and 80%	Showing green for 3 min	There were no significant differences in physiological parameters with respect to the green index. Significant physiological and psychological improvements. Subjectively, participants preferred 50% of the green index the most.
Johan Ottosson and Patrik Grahn (2005) [38]	Sweden	Randomized controlled trials	N = 15 (2 males and 13 females) Mean age: 86 Elderly people living in homes for the elderly.	Greenery included: Elderly home garden (experimental) No greenery: Elderly home indoor (control)	Outdoor leisure activities 1 h	Did not show any effect on blood pressure or heart rate. However, there was a restoration of attention in the elderly.
K. Dijkstra et al., (2008) [36]	Netherlands	Randomized controlled trials	N = 77 (35 males and 42 females) Mean age: 21, SD = 2.2; Students, no details	Greenery included: With plant ward photo (experimental) No greenery: Without plant ward photo (control)	View photos of hospital rooms (exact time unknown)	Participants in the ward with indoor plants felt less self-reported stress than those in the ward with paintings. Indoor plants reduced stress by increasing the attractiveness of the room.
Kate E. Lee et al., (2015) [35]	Australia	Randomized controlled trials	N = 150 (71% females) Mean age: 20 Volunteers were recruited from the University Psychology Research Experience Program and the broader student population.	Greenery included: Green roof simulation view (experimental) No greenery: Urban scenario with a concrete roof (control)	View 40 s	Changes in subcortical arousal and cortical attentional control occur. Attention is restored, and subsequent benefits may include improved performance and mood, as well as reduced stress.
Katinka H. Evensen1 et al., (2013) [42]	Norway	Randomized controlled trials	N = 85 (28 males and 57 females) Mean age: 24.9, SD = 5.7 College students	Greenery included: Room with plants with windows (experimental); room with plants without windows (experimental) No greenery: Rooms with computers only (control)	Oriented attention for 10 min	There was no significant restorative effect of plants. Visual richness in the environment may be a restorative factor. The presence of indoor plants led to higher levels of perceptual fascination with the environment.
Ke-Tsung Han (2008) [43]	China	Non-randomized control trials	N = 76 (58 males and 18 females) Mean age: 13.6 (experimental group); 13.5 (control group) Two classes of students in the second year of junior high school	Greenery included: Cinnamomum kotoense in the classroom (experimental) No greenery: No plants in the classroom (control)	Exposure to plant environment for 2.5 months	There was an immediate stronger sense of preference, comfort, and friendliness in the experimental group compared to the control group. STAI, RCS, and RS increased over time, and WBM decreased over time, with improvements over time but not statistically significant.
Lee, MS (Lee, Min-sun) et al., (2015) [46]	Korea	Randomized controlled trials	N = 24 (all males) Mean age: 24.9, SD = 2.1 Young male adults	Greenery included: Caring for houseplants (experimental) No greenery: Computer tasks (control)	15 min plant transplanting work	Mean activity of HRV increased over time and decreased at the end of the plant task. Diastolic blood pressure decreased. Positive interaction with houseplants reduces physical and psychological stress.
Masahiro Toyoda et al., (2019) [37]	Japan	Non-randomized control trials	N = 63 (33 males and 30 females) Mean age: 38.7, SD = 9.3(male); 41.6, SD = 9.6 (female) Electricity company employees.	Greenery included: Small plants on the desk (experimental) No greenery: No plants on the desk (control)	Observe plants and take care of them for 4 weeks	STAI scores decreased significantly after the intervention period (*p* < 0.05); pulse rate decreased throughout. Conscious gazing at nearby plants can reduce psychological and physical stress in office workers.
Preyen Archary and Andrew Thatcher (2021) [41]	USA	Randomized controlled trials	N = 60 (21 males and 39 females) Mean age: 21.80, SD = 6.09 Undergraduate students	Greenery included: Room with two large indoor foliage plants and one small bonsai table plant (experimental) No greenery: Facing the wall with no plants or other irritants (control)	Facing the plant for 6 min	Distress was significantly reduced, and engagement increased. The presence of indoor plants had a small positive effect on affective recovery and no effect on cognitive recovery.
Seong-Hyun Park and Richard H. Mattson (2008) [48]	USA	Randomized controlled trials	N = 90 (52 males and 38 females) Mean age: 37.6, SD = 9.41 Patients who had undergone an appendectomy	Greenery included: Ward with foliage and flowering plants, 12 potted foliage and flowering plants (experimental) No greenery: No plants in the ward (control)	Mean length of stay in wards with plants 4.64 days	Patients had significantly lower postoperative analgesic intake and more positive physiological responses (as evidenced by lower systolic blood pressure and heart rate). Pain, anxiety, and fatigue scores were lower.
Seungkeun Yeom et al., (2021) [16]	Korea	Randomized controlled trials	N = 27 (17 males and 10 females) Mean age: 25.53 (male); 23.15 (female) College students of different majors at Yonsei University	Greenery included: 8.0 m^2^ indoor virtual big green wall (experimental); 2.0 m^2^ indoor virtual small green wall (experimental) No greenery: Blank wall (control)	Sit in a chair for 5 min and walk around for 1 min	The small green wall had a more dramatic improvement effect. Subjects in the small green wall condition had substantially lower stress levels compared to the non-green wall condition. The large green wall reduced STAI levels but increased mental stress, fatigue, and anxiety.
Virginia I. Lohr et al., (1996) [49]	USA	Non-randomized control trials	N = 96 (48 males and 48 females) Age: 18–46 (No average age information) Predominantly from undergraduate agricultural economics classes	Greenery included: Computer labs with plants (experimental) No greenery: Computer lab without plants (control)	Computer tasks completed in indoor spaces with plants	Participants were more productive. Less stressful and more focused. Significant increase in attention span.
WeiLin et al., (2019) [12]	China	Randomized controlled trials	N = 240 (53% females) Mean age: 20.2, SD = 1.76	Six different types of small simulated green spaces	Exposure of green space for 10 min	Walking in high PCA (per capita area) and sitting in low PCA have the most powerful effect on reducing stress

Abbreviations: HRV: Heart Rate Variability; HR: Heart Rate; EEG: Electroencephalogram; EDA: Electrodermal activity; PRS: Perceptual Recovery Scale; STAI: State Trait Anxiety Inventory; DSSQ-S: Dundee Stress State Questionnaire; RCS: Restorative Components Scale; RS: Restorative Scale; TSST: Trier Social Stress Test; POMS: Profile of Mood States; ZIPER: Zuckerman Inventory of Personal Reactions; TMD: Total Mood Disorder; SD: Semantic Differences.

**Table 3:** Interventions and effectiveness results.

Greening Type	Authors and Year of Publication	Physiological Stress Indicator	Psychological Stress Indicators	Results
Indoors	A. Hassan et al., (2020) [39]	Blood Pressure; EEG	State Trait Anxiety Inventory (STAI)	Stress has been significantly reduced
	Hassan, Ahmad et al., (2018) [47]	Blood Pressure; EEG	SDM; STAI	
	H. Ikei et al., (2014) [40]	HRV; Pulse	SD	
	Jiang S et al., (2021) [45]	Blood Pressure; Pulse; EEG	POMS; TMD; SD	
	Ji-Young Choi et al., (2016) [50]	HRV; EEG	SD	
	Ke-Tsung Han (2008) [43]		STAI; RCS; RS	
	Lee, MS (Lee, Min-sun) et al., (2015) [46]	HRV; Blood Pressure	SDM	
	Masahiro Toyoda et al., (2019) [37]	Pulse	STAI	
	Seong-Hyun Park and Richard H. Mattson (2008) [48]	Blood Pressure; HR	STAI	
	Virginia I. Lohr et al., (1996) [49]	Blood Pressure; Pulse	ZIPER	
	Katinka H. Evensen1 et al., (2013) [42]		PRS; Likert Scale	No effect on pressure is shown
	Preyen Archary and Andrew Thatcher (2021) [41]		DSSQ-S	
	WeiLin et al., (2019) [12]	Blood Pressure; Pulse; Finger SpO2	TMD; POMS	Stress has been significantly reduced
Outdoors	Johan Ottosson and Patrik Grahn (2005) [38]	Systolic Blood Pressure; Diastolic Blood Pressure; HR		No effect on pressure is shown
Virtual	Kate E. Lee et al., (2015) [35]		6-point Likert scale; PRS	Stress has been significantly reduced, but the big green wall may add pressure
	Seungkeun Yeom et al., (2021) [16]	HR; EDA; EEG	STAI;	
Photos	K. Dijkstra et al., (2008) [36]		Stress Arousal Checklist	Stress has been significantly reduced
Photos or Indoors	Cammie K. Coleman and Richard H. Mattson (1995) [44]	Skin Temperature		Live plants and photographs had a positive response for 38% of participants, and 23% had reduced stress in the control group
Video	Bin Jiang et al., (2014) [5]	Salivary Cortisol; Skin Conductance	TSST	Stress has been significantly reduced

Abbreviations: HRV: Heart Rate Variability; HR: Heart Rate; EEG: Electroencephalogram; EDA: Electrodermal activity; PRS: Perceptual Recovery Scale; STAI: State Trait Anxiety Inventory; DSSQ-S: Dundee Stress State Questionnaire; RCS: Restorative Components Scale; RS: Restorative Scale; TSST: Trier Social Stress Test; POMS: Profile of Mood States; ZIPER: Zuckerman Inventory of Personal Reactions; TMD: Total Mood Disorder; SD: Semantic Differences.

## Data Availability

Data is contained within this manuscript or in the Supplementary Materials.

## Supplementary Materials

The following supporting information can be downloaded at: https://www.mdpi.com/article/10.3390/ijerph19169778/s1, Supplementary Material S1: PRISMA Checklists; Supplementary Material S2: Search strategies.

## Appendix A

Risk of bias tables for included RCT studies using the Cochrane Collaboration Assessment Tool and for non-RCT included studies using the Risk of Bias Assessment Tool for Non-Randomized Studies (RoBINS-I).

**Table A1:** ROBINS-I assessments.

	Confounding	Participant Selection	Classification of Interventions	Deviation from Intended Intervention	Missing Data	Measurement of Outcomes	Reporting Bias	Overall Bias
A. Hassan et al., (2020)	Moderate	Low	Low	Low	Low	Moderate	Moderate	Moderate
Cammie K Coleman and Richard H. Mattson (1995)	Serious	Low	Low	Low	Serious	Serious	Moderate	Serious
Hassan, Ahmad et al., (2018)	No information	Low	Low	Low	Low	Serious	Moderate	Serious
Ke-Tsung Han (2008)	Serious	Low	Low	Low	Low	Serious	Moderate	Serious
Masahiro Toyoda et al., (2019)	Serious	Low	Low	Moderate	Low	Moderate	Moderate	Serious
Virginia I. Lohr et al., (1996)	Serious	Low	Low	Low	Low	Serious	Moderate	Serious
