# Supplementary material for: Can Even a Small Amount of Greenery Be Helpful in Reducing Stress? A Systematic Review
Source: Int J Environ Res Public Health. 2022 Aug 9;19(16):9778. doi: 10.3390/ijerph19169778 (PMC9408062; doi:10.3390/ijerph19169778)
Supplement: Supplementary file 1 [file ijerph-19-09778-s001.zip › ijerph-1823744-supplementary/supplementary material S2.pdf]

S2: Search strategies for all databases searched

Embase

(participants OR 'students'/exp OR students OR subjects OR 'volunteer'/exp OR volunteer OR people OR respondents) AND ('pocket parks' OR 'small-scale parks' OR 'small parks' OR 'mini-parks' OR 'small green spaces' OR 'tiny green space' OR 'small green area' OR 'tiny parks' OR parkland OR 'residential green space' OR 'roof garden' OR 'home garden' OR 'green roof'/exp OR 'green roof' OR 'street green space' OR 'campus green*' OR 'street green*' OR 'community green*' OR 'natural environment'/exp OR 'natural environment' OR 'natural outdoor environment' OR 'indoor plants') AND ('intervention'/exp OR intervention OR 'experiment'/exp OR experiment OR 'randomized controlled trial'/exp OR 'randomized controlled trial' OR randomized OR crossover OR 'case-crossover' OR 'pre-post' OR 'comparison'/exp OR comparison OR 'non randomized' OR 'exposure'/exp OR exposure OR controlled OR 'control'/exp OR control OR 'control group'/exp OR 'control group') AND ('stress reduct*' OR 'stress relief' OR 'stress-related' OR 'restoration'/exp OR restoration OR 'stress'/exp OR stress OR 'well-being'/exp OR 'well-being' OR 'physiological benefits' OR 'psychological traits' OR 'wellbeing'/exp OR wellbeing OR 'psychological outcome' OR 'emotion*' OR 'mood'/exp OR mood OR 'blood pressure'/exp OR 'blood pressure' OR 'mental health'/exp OR 'mental health')

Pubmed

(((participants[Title/Abstract] OR 'students[Title/Abstract] OR subjects[Title/Abstract] OR volunteer[Title/Abstract] OR people[Title/Abstract] OR respondents[Title/Abstract]) AND ("pocket parks"[Title/Abstract] OR "small-scale parks"[Title/Abstract] OR "small parks"[Title/Abstract] OR "mini-parks"[Title/Abstract] OR "small green spaces"[Title/Abstract] OR "tiny green space"[Title/Abstract] OR "Small green area"[Title/Abstract] OR "tiny parks"[Title/Abstract] OR parkland[Title/Abstract] OR "Residential Green Space"[Title/Abstract] OR "Roof garden "[Title/Abstract] OR "Home Garden"[Title/Abstract] OR "Green Roof"[Title/Abstract] OR "Street green space"[Title/Abstract] OR "Campus Green*"[Title/Abstract] OR "Street Green*"[Title/Abstract] OR "Community Green*"[Title/Abstract] OR "natural environment"[Title/Abstract] OR "natural outdoor environment"[Title/Abstract] OR " indoor plants"[Title/Abstract])) AND (intervention[Title/Abstract] OR experiment[Title/Abstract] OR "randomized controlled trial"[Title/Abstract] OR randomized[Title/Abstract] OR crossover[Title/Abstract] OR "case-crossover"[Title/Abstract] OR pre-post[Title/Abstract] OR comparison[Title/Abstract] OR non-randomized[Title/Abstract] OR exposure[Title/Abstract] OR controlled[Title/Abstract] OR control[Title/Abstract] OR "control group"[Title/Abstract])) AND ("stress reduct*"[Title/Abstract] OR "stress relief"[Title/Abstract] OR "stress-related"[Title/Abstract] OR restoration[Title/Abstract] OR stress[Title/Abstract] OR "well-being"[Title/Abstract] OR "physiological benefits"[Title/Abstract] OR "psychological traits"[Title/Abstract] OR wellbeing[Title/Abstract] OR "psychological outcome"[Title/Abstract] OR "emotion*"[Title/Abstract] OR mood[Title/Abstract] OR "blood pressure"[Title/Abstract] OR "mental health"[Title/Abstract])

Scopus

TITLE-ABS-KEY (participants OR students OR subjects OR volunteer OR people OR respondents) AND TITLE-ABS-KEY ("pocket parks" OR "small-scale parks" OR "small parks" OR "mini-parks" OR "small green spaces" OR "tiny green space" OR "Small green area" OR "tiny parks" OR parkland OR "Residential Green Space" OR "Roof garden " OR "Home Garden" OR "Green Roof" OR "Street green space" OR " indoor plants" OR "Campus Green*" OR "Street Green*" OR "Community Green*" OR "natural environment" OR "natural outdoor environment") AND TITLE-ABS-KEY (intervention OR

experiment OR "randomized controlled trial" OR randomized OR crossover OR "case-crossover" OR pre-post OR comparison OR non-randomized OR exposure OR controlled OR control OR "control group") AND TITLE-ABS-KEY ("stress reduct*" OR "stress relief" OR "stress-related" OR restoration OR stress OR "well-being" OR "physiological benefits" OR "psychological traits" OR wellbeing OR "psychological outcome" OR "emotion*" OR mood OR "blood pressure" OR "mental health")

Science direct

('small green' OR "Green Roof" OR "Street green' OR "small park" OR " indoor plants") AND("mental health" OR "stress reduction" OR restoration OR psychological)

WOS

((((TS=(participants OR 'students OR subjects OR volunteer OR people OR respondents)) AND TS=("pocket parks" OR "small-scale parks" OR "small parks" OR "mini-parks" OR "small green spaces" OR "tiny green space" OR "Small green area" OR "tiny parks" OR parkland OR "Residential Green Space" OR "Roof garden " OR "Home Garden" OR "Green Roof" OR "Street green space" OR "Campus Green*" OR "Street Green*" OR "Community Green*" OR "natural environment" OR "natural outdoor environment"OR " indoor plants")) AND TS=(intervention OR experiment OR "randomized controlled trial" OR randomized OR crossover OR "case-crossover" OR pre-post OR comparison OR non-randomized OR exposure OR controlled OR control OR "control group")) AND TS=("stress reduct*" OR "stress relief" OR "stress-related" OR restoration OR stress OR "well-being" OR "physiological benefits" OR "psychological traits" OR wellbeing OR "psychological outcome" OR "emotion*" OR mood OR "blood pressure" OR "mental health"))

Cochrane

- participants OR 'students OR subjects OR volunteer OR people OR respondents
- "pocket parks" OR "small-scale parks" OR "small parks" OR "mini-parks" OR "small green spaces" OR "tiny green space" OR "Small green area" OR "tiny parks" OR parkland OR "Residential Green Space" OR "Roof garden " OR "Home Garden" OR "Green Roof" OR "Street green space" OR "Campus Green*" OR "Street Green*" OR "Community Green*" OR "natural environment" OR "natural outdoor environment" OR " indoor plants"
- intervention OR experiment OR "randomized controlled trial" OR randomized OR crossover OR "case-crossover" OR pre-post OR comparison OR non-randomized OR exposure OR controlled OR control OR "control group"
- "stress reduct*" OR "stress relief" OR "stress-related" OR restoration OR stress OR "well-being" OR "physiological benefits" OR "psychological traits" OR wellbeing OR "psychological outcome" OR "emotion*" OR mood OR "blood pressure" OR "mental health"
